# The Implementation and One-Year Evaluation of a Pre-Admission Pharmacist for Obstetric Admissions

**DOI:** 10.3390/pharmacy14050119

**Published:** 2026-08-17

**Authors:** Bridget Clark, Nabeelah Mukadam, Tamara Lebedevs, Stephanie Wai Khuan Teoh

**Affiliations:** Women and Newborn Health Service, King Edward Memorial Hospital, Subiaco 6008, Australia; bridget.clark@health.wa.gov.au (B.C.); nabeelah.mukadam@health.wa.gov.au (N.M.); tamara.lebedevs@health.wa.gov.au (T.L.)

**Keywords:** medication history, pre-admission clinic, obstetric and gynaecology, telehealth consultation

## Abstract

(1) Objective: This study aims to evaluate the pre-admission clinic (PAC) pharmacist for high-risk obstetric patients’ admissions on medication management processes, workflow efficiency, and staff satisfaction. (2) Methods: Data collected over a 12-month period, from March 2025 to February 2026, were analysed. Data collected included clinic type, patient attendance, mode of pharmacist review, and follow-up requirements. Multidisciplinary healthcare professionals were invited to complete a satisfaction survey to identify themes relating to service value, workflow impact, and opportunities for improvement. (3) Results: A total of 1039 interviews with obstetric patients, equating to an average of 19.98 interviews per week. A total of 999 patients were involved in the 1039 interviews held, 40/999 (4%) patients required additional follow-up telephone interview and 14 patients (14/999, 1.4%) required in-person interview for interpreter services, detailed discussion or hearing impairment. Thirty-two staff responded to a survey and demonstrated a consistent trend across anaesthetists, nursing and midwifery staff, and pharmacists. All respondents either agreed or strongly agreed that a PAC pharmacist in the obstetric setting was valuable and beneficial to clinical workflow and medication management processes, and that they were satisfied with the PAC pharmacy service. (4) Conclusions: The PAC pharmacist demonstrated a positive impact on medication management for obstetric patients within the pre-admission setting.

## 1. Introduction

Medication safety in pregnancy requires careful consideration of both maternal and foetal wellbeing. Physiological changes during pregnancy, increasing rates of multimorbidity, and the increasing prevalence of high-risk obstetric populations contribute to heightened vulnerability to medication-related harm [1]. Ensuring accurate medication histories and timely optimization of pharmacotherapy in this cohort of patients is critical. Medication discrepancies and delayed medication reconciliation remain common in both obstetric and general hospital settings, with potential consequences including adverse drug events, delayed treatment and suboptimal therapeutic outcomes [2,3].

Pharmacist-led interventions, including medication reconciliation and clinical review, have consistently demonstrated improved medication safety and reduced errors across healthcare settings. Clinical pharmacist services are associated with improved accuracy of best possible medication histories (BPMHs), enhanced identification of drug-related problems, and more timely provision of clinical recommendations [4,5]. While some studies support embedded ward-based pharmacists as sufficient, others report that dedicated pre-admission or outpatient pharmacy clinics offer improved efficiency, earlier intervention, and better continuity of care [2,3,6,7,8,9].

Proactive medication review has been associated with optimized pharmacotherapy regimens, enhanced multidisciplinary communication, and potential reductions in medication-related problems. Within tertiary hospitals, pre-admission clinics (PACs) provide a critical opportunity to identify and address medication-related problems prior to admission [6,7,8,9,10,11,12,13,14,15]. Previous studies have demonstrated the importance of the PAC pharmacist in reducing medication-related problems through improved quality of BPMH documentation, medication reconciliation, and pharmacist intervention [6,7,8,9,10,11,12,13,14,15]. The writing of discharge prescriptions transcribing prescription orders was suggested to be a valuable service provided by the PAC pharmacist, while it was also recognized that extra resources would be needed in order to provide the service [12,13].

Within obstetric care, the integration of pharmacists into pre-admission workflows remains variable, while the evidence that supports the use of dedicated pharmacist-led clinics for high-risk obstetric patients is still emerging. Recent literature highlights the value of early pharmacist involvement in antenatal and pre-admission care, particularly for patients with complex comorbidities, polypharmacy, or high-risk conditions such as diabetes, hypertension, and thromboembolic disease [2,3,4,5]. The implementation of a pharmacist-led pharmacy review clinic within this high-risk setting facilitates comprehensive medication management and medication review during a vulnerable period in a woman’s pregnancy journey [16,17]. A recent study evaluating the impact of a pharmacist-led elective caesarean section PAC demonstrated enhance workflow efficiency, BPMH completion and increased patient preparedness for post-operative analgesic supply on discharge [11].

At the study hospital, pharmacy services have supported the assessment of high-risk patients in three of six pre-admission clinics since 2007, within the gynaecology, urogynaecology and gynaecological oncology surgical clinics [18]. Historically, clinical pharmacist advice was provided on an ad hoc basis through pharmacy intern support when required, averaging approximately 6–7 patients per week [18]. In 2025, a dedicated PAC pharmacist role was formalized across the hospital’s pre-admission services, with the scope of the role expanded to include all six PACs. This expansion incorporated elective caesarean section, high-risk anaesthetic, and high-risk obstetric PACs, in addition to the existing gynaecological surgical services. The establishment of this role aimed to improve consistency of medication management, enhance multidisciplinary support, and strengthen medication safety processes for high-risk surgical and obstetric patients.

This study aims to investigate the impact of a pharmacist-led pharmacy review clinic for high-risk obstetric patients on multidisciplinary workflow efficiency and medication management processes. It seeks to determine whether the introduction of a pharmacist-generated medication list with documented advice and recommendations improves efficiency, preparedness, and staff satisfaction. Finally, it aims to assess if this service helps with medication charting on admission and enhances overall efficiency across the multidisciplinary team.

## 2. Materials and Methods

Patients identified as high-risk were contacted by the PAC pharmacist prior to their scheduled in-person PAC appointment. Six PACs were conducted weekly at the study hospital, The obstetric PAC pharmacy service consisted of 3 weekly clinics, including 2 pre-elective caesarean clinics and 1 high-risk anaesthetic obstetric clinic. The main mode of communication for pharmacy PAC was through telephone interview. The patients were contacted by the PAC pharmacist by phone a week prior to their scheduled in-person PAC appointment. A control group was not considered, as this project is a continuation and formalisation of existing patient care at the pre-admission clinic, with a dedicated PAC pharmacist role formalized across the hospital’s pre-admission services, instead of on ad hoc basis.

During the consultation, the pharmacist obtained a comprehensive BPMH using multiple information sources to maximize completeness and accuracy. Sources included patient interview, dispensing histories, digital medical record (DMR), a statewide medical record review, referral documentation, and communication with community healthcare providers where required. The pharmacist also screened medications for preoperative considerations, including the need for withholding, continuation, or alteration prior to surgery, and assessed for potential interactions with commonly used anaesthetic agents. The BPMH is documented on the medication list for PAC form, details documented include medication generic name/trade name, strength and form (i.e., slow release, wafers, etc.), dose, frequency and route, withhold prior to surgery (yes/no), and special instructions. Where additional support or clarification was required, such as interpreter services, hearing impairment, or uncertainty regarding medication regimens, the consultation was conducted in person during the patient’s scheduled PAC appointment.

During the consultation, the pharmacist provided individualized medication instructions to patients and, where necessary, liaised with community dose administration aid-packing pharmacies to facilitate pre-operative medication changes. Following review, a pharmacist-generated medication list (medication list for pre-admission) and written medication advice were completed. The written advice included instructions regarding medication changes required prior to surgery, as well as reminders for patients to bring their usual medications to hospital on admission. This written advice process was implemented as a quality improvement initiative following recommendations from staff feedback obtained in a previous service evaluation [12].

**Figure 1:**
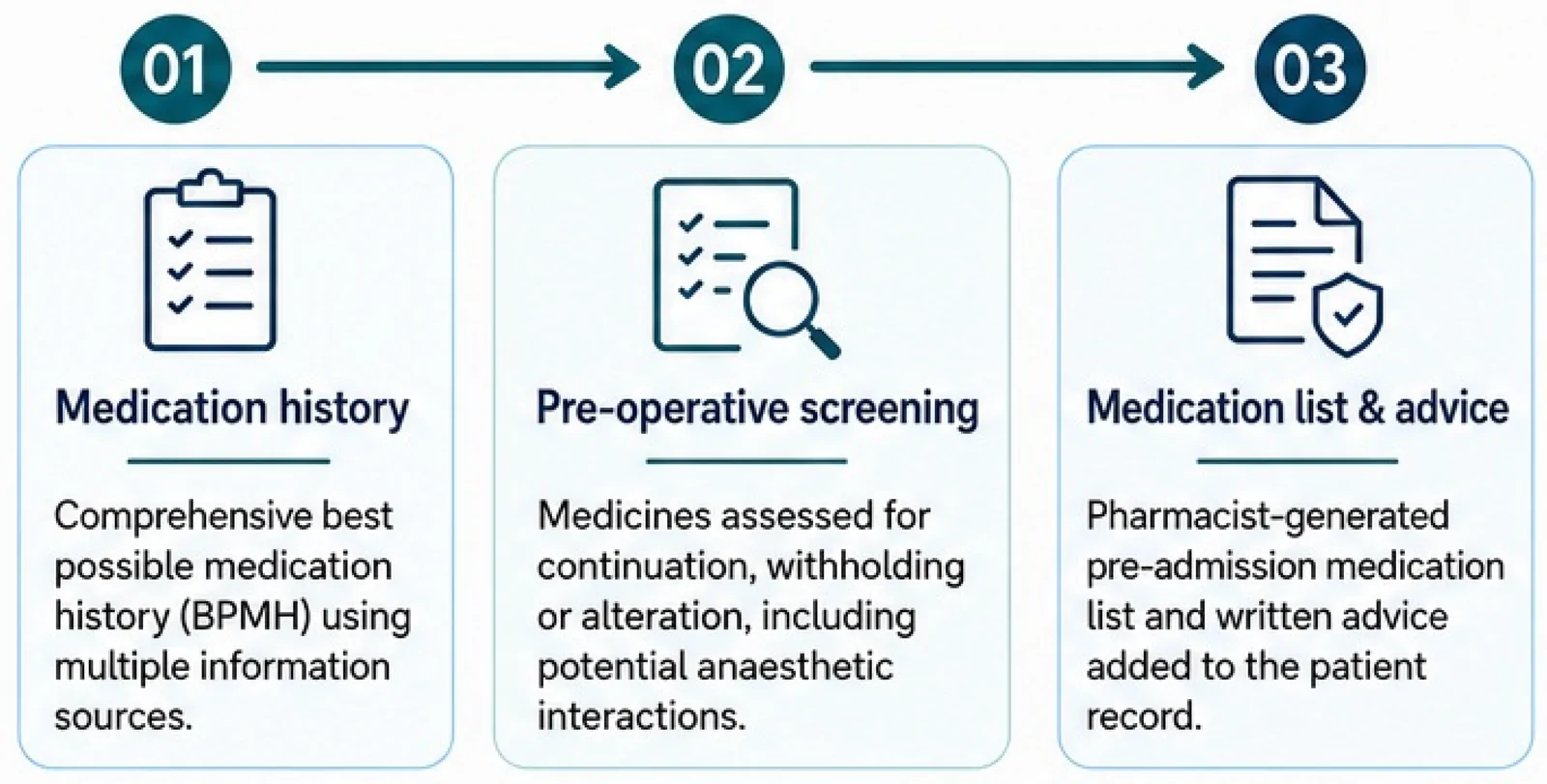
Workflow of a PAC pharmacist. During the consultation, the pharmacist obtained a comprehensive BPMH using multiple information sources to maximize completeness and accuracy. The pharmacist also screened medications for preoperative considerations, including the need for withholding, continuation, or alteration prior to surgery, and assessed for potential interactions with commonly used anaesthetic agents. Following the PAC pharmacist consultation, both the medication list and written advice were uploaded to the patient’s medical record to ensure accessibility by the multidisciplinary team.

Following the PAC pharmacist consultation, both the medication list and written advice were uploaded to the patient’s DMR to ensure accessibility by the multidisciplinary team. In addition, a physical copy was included in the patient’s surgical pack for reference during the PAC appointment and admission process. The workflow of the PAC pharmacist is summarized in Figure 1.

### 2.1. Data Collection Method

Data collected over a 12-month period, from March 2025 when the PAC pharmacist expanded the service to obstetric patients, to February 2026, were analysed in this study. Key patient details, including unique medical record number, date of admission, and date of interview, clinic type, patient attendance, mode of pharmacist review (telephone or client present), and follow-up requirements, were recorded in a secured Microsoft Excel^®^ spreadsheet, accessible by the PAC pharmacist and the deputy chief pharmacist. This spreadsheet was used to support service activity tracking, record pharmacist review activity, and document attendance in the hospital patient administrative system.

### 2.2. Staff Survey Method

Multidisciplinary healthcare professionals including medical, nursing, midwifery, and allied health staff at PAC, and all clinical pharmacists were invited to complete an online satisfaction survey via Microsoft Forms^®^ from February to April, 2026. Non-clinical staff were excluded.

The survey included statements seeking level of agreement and an overall rating of satisfaction using the response options across a 5-point Likert scale of strongly agree to strongly disagree. Nurses, midwives and allied health staff received the same questions, while anaesthetists and pharmacists received additional questions specific to their roles. Health professionals were also invited to provide free-text recommendations and comments. The questionnaire in the survey was not validated and was designed based on the previous staff survey used with more specific questions for pharmacists and anaesthetists [18].

Survey responses were collated and analysed using Microsoft Excel^®^. Descriptive statistics were used to summarize responses, including frequences and percentages. Free-text responses were reviewed to identify recurring themes relating to service value, workflow impact, communication, and opportunities for improvement.

Human research ethics approval was gained from the Women and Newborn Health Service Quality Improvement Committee (approval number: GEKO 64450) at the study hospital.

## 3. Results

### 3.1. Data Collection

**Figure 2:**
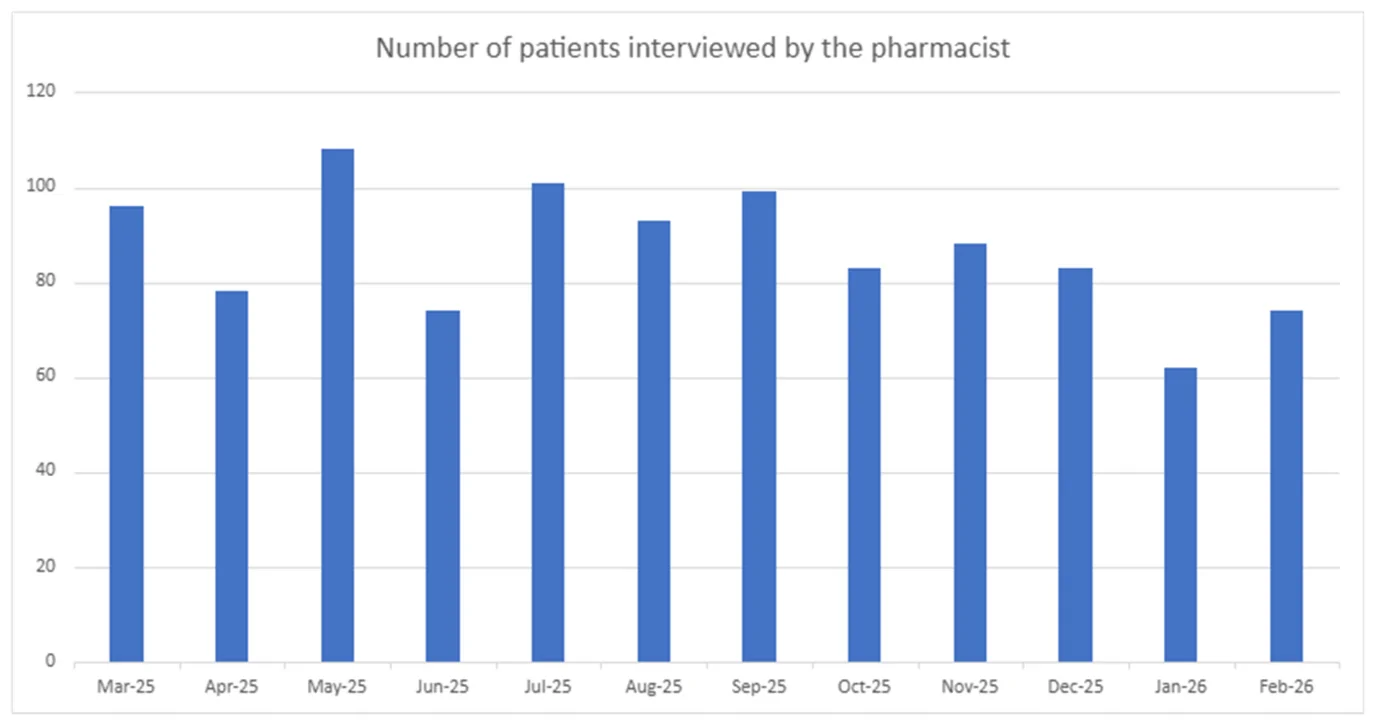
Number of patients interviewed by the PAC pharmacist from March 2025 to February 2026.

Service activity data were prospectively collected over a 12-month period for patients reviewed through the obstetric pre-admission clinics (PACs). During the study period, a total of 1039 interviews with obstetric patients were held by the PAC pharmacist, equating to an average of 19.98 interviews per week (Figure 2). A total of 999 patients were involved in the 1039 interviews held, with 40/999 (4%) patients required additional follow-up telephone interview following initial review. The follow-up telephone consultation was organized to clarify medication histories, provide further medication counselling, confirm preoperative medication management plans, or communicate medication changes. A total of 14 patients (14/999, 1.4%) required additional support, including those with language barriers requiring interpreter services (2/999, 0.2%), those with complex medication regimens requiring detailed discussion, or those with hearing impairment requiring further clarification (12/999, 1.2%). These patients were reviewed in person during their scheduled in-person PAC appointment.

Out of 999 patients interviewed by the PAC pharmacists, 775/999 (77.6%) had their planned admission date confirmed at the time of interview. Reasons for unknown admission date include spontaneous vertex delivery, extended time between PAC and approximate admission week and early referral to PAC pharmacist during antenatal care. Of the 775 patients with a known admission date, the average days between PAC and admission date was 12.4 days, and the median was 13 days.

### 3.2. Staff Survey

**Figure 3:**
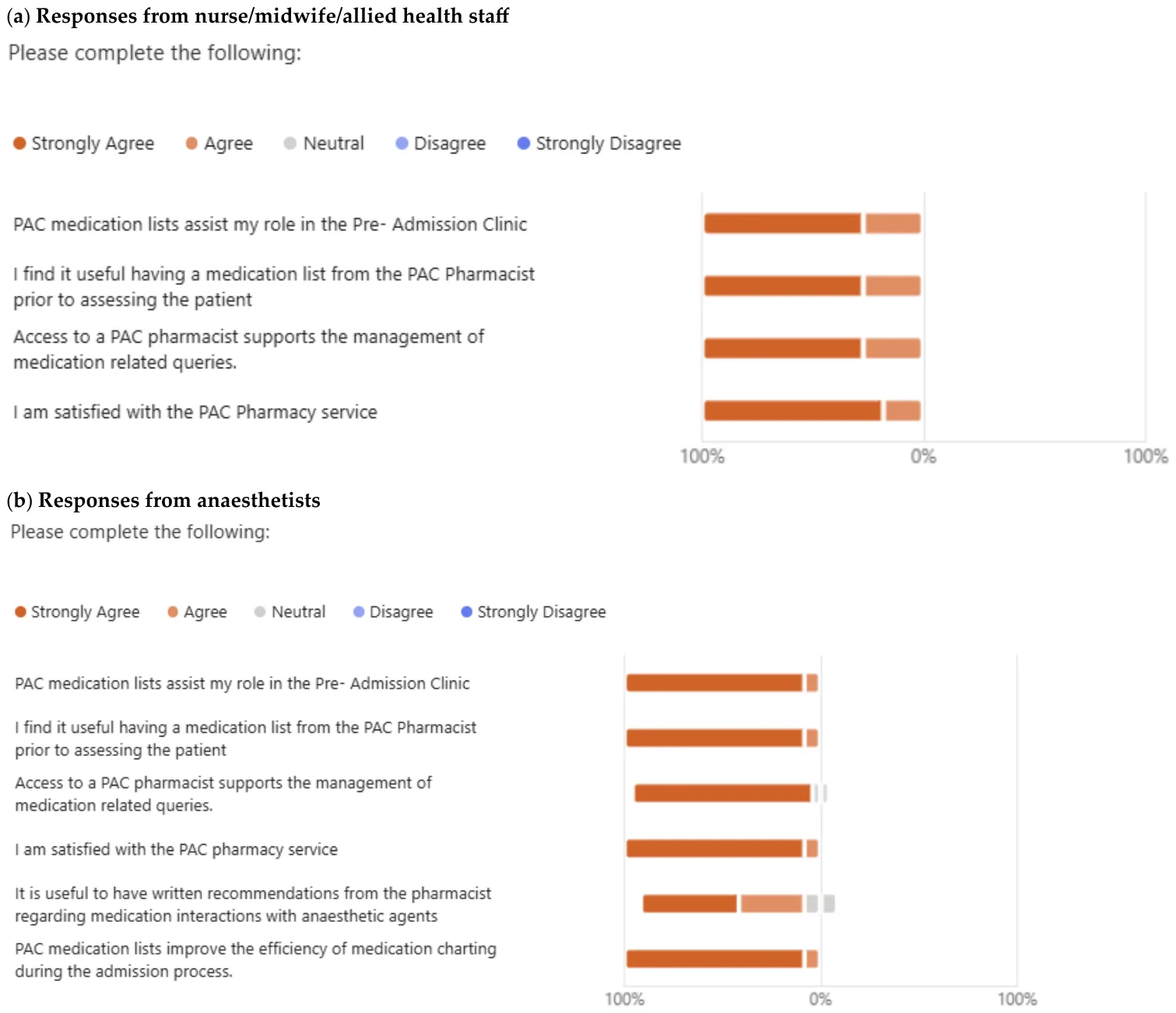

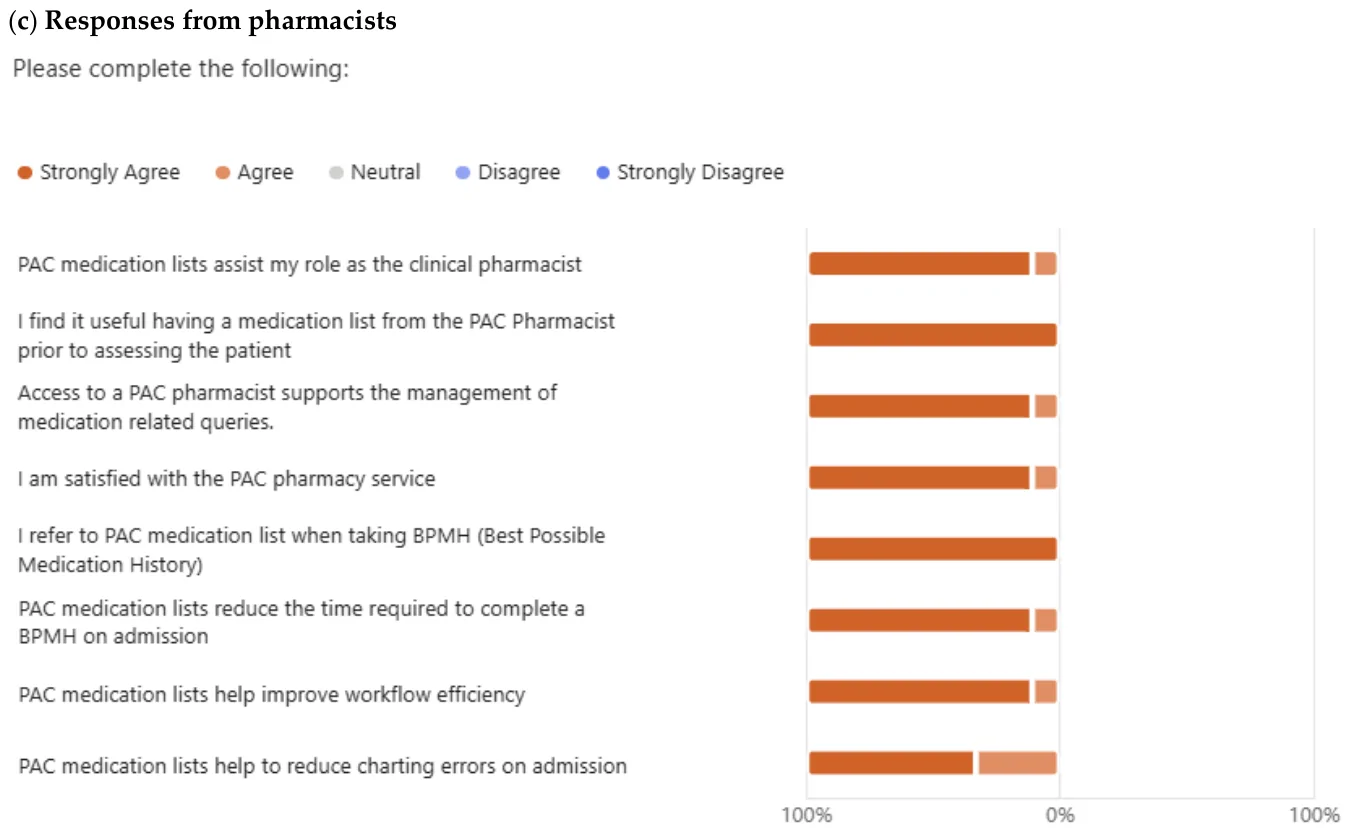
Responses from staff survey (**a**) nurse/midwife/allied health, (**b**) anaesthetists, (**c**) pharmacists.

A total of 32 survey responses were received out of approximately 50 multidisciplinary staff at PAC and clinical pharmacists, representing a 64% response rate. Responders were 12 anaesthetists, 10 nurses/midwives, nine pharmacists, and one allied health staff member. Among 11 nurses, midwives, and allied health staff respondents, 8/11 (72.7%) strongly agreed and 3/11 (27.3%) agreed that the medication list generated by the PAC pharmacist assisted in their role and was useful to have prior to patient assessment and that access to a PAC pharmacist supported the management of medication-related queries. Additionally, 9/11 (81.8%) strongly agreed and 2/11 (18.2%) agreed that they were satisfied with the PAC pharmacy service overall (Figure 3).

Of the 12 responses received from anaesthetists, 11 respondents identified as anaesthetic consultants, while 1 respondent did not specify their role (Figure 2). When asked how frequently they had utilized the PAC pharmacy service including reviewing pharmacist-generated medication lists or documented advice or directly consulting with the PAC pharmacist regarding patient management, one respondent did not provide a response, 3/12 (25%) reported utilizing the service between 6 and 10 times, and 8/12 (66.7%) indicated they had used the service more than 10 times.

A total of 11/12 (91.7%) strongly agreed and 1/12 (8.3%) agreed that they were satisfied with the PAC pharmacy service. Respondents also agreed that the medication list generated by the PAC pharmacist assisted in their role, was useful to have prior to patient assessment, and improved the efficiency of medication charting during the admission process. Regarding the usefulness of written pharmacist recommendations related to medication interactions with anaesthetic agents, 6/12 (50%) strongly agreed, 4/12 (33.3%) agreed, and 2/12 (16.7%) were neutral.

Nine responses were received from pharmacists. All pharmacist respondents (100%) strongly agreed that having access to a medication list generated by the PAC pharmacist prior to patient assessment was useful and that they referred to the PAC medication list when completing the BPMH (Figure 2).

Additionally, 8/9 (88.9%) strongly agreed and 1/9 (11.1%) agreed that PAC medication lists assisted their role as a clinical pharmacist, that access to a PAC pharmacist supported the management of medication-related queries, and that they were satisfied with the PAC pharmacy service. Regarding medication safety outcomes, 6/9 (66.7%) strongly agreed, and 3/9 (33.3%) agreed that PAC medication lists contributed to a reduction in charting errors on admission.

When asked to leave a free-text response on areas for improvement for the PAC pharmacy service, the responses received were very positive. Some of the responses received are highlighted in Table 1.

## 4. Discussion

This one-year evaluation examined the introduction of the PAC pharmacy service within the obstetric directorate and its impact on pharmacist-led medication review for obstetric patients. During the study period, 959/999 (96%) of patients did not require follow-up interviews and 999/1039 (98.6%) of interviews were conducted by telephone. The documented number of patients interviewed was slightly higher than that of a recent study, which reported 162 patients interviewed face-to-face in 3 months; however, this is difficult to compare due to different the PAC setting, the mode of interview, and the study design [10]. Our study also suggested that telephone consultation was effective for obtaining BPMHs and providing medication advice for most obstetric patients. A study conducted in an elective procedure admission clinic demonstrated that medication history interviews undertaken via telephone are a suitable alternative to face-to-face medication history-taking in low-risk elective surgical patients who are unable or not required to attend an elective procedures admission clinic [11]. The provision of clinical pharmacist service via telephone has been reported in several studies and the services included the outpatient or ambulatory care setting, opioid risk assessment clinic, and oral chemotherapy clinic [19,20,21,22].

The established workflow of the PAC pharmacist, in which the pharmacist completed a telephone consultation approximately one week prior to the patient’s scheduled PAC appointment with nursing and anaesthetists was well received by the multidisciplinary team. This process ensured that, prior to the patient’s attendance of a PAC, the pharmacist-generated medication list and written medication advice were available in the patient’s medical file, which was accessible by anaesthetists, nurses, midwives, and allied health staff. Prior to clinical assessment, the availability of this information supported more consistent admission planning and improved communication across the multidisciplinary team, and contributed to greater workflow efficiency within the PAC setting.

The multidisciplinary staff survey responses demonstrated a consistent trend across anaesthetists, nursing and midwifery staff, and pharmacists in which pharmacist involvement within the PAC setting was valuable and beneficial to clinical workflow and medication management processes. Free-text responses further supported these findings, with staff frequently describing the PAC pharmacist role as highly valued and beneficial to patient care and multidisciplinary communication. No respondents selected “disagree” or “strongly disagree” for any survey statement relating to the usefulness or satisfaction with the PAC pharmacy service. All respondents either agreed or strongly agreed that they were satisfied with the PAC pharmacy service. Compared with the staff survey conducted in 2022, these findings suggest improved staff satisfaction and perceived value following the formalization and expansion of the PAC pharmacist role [18]. No other staff satisfaction study was located with PAC pharmacy services, and one other study reported good patient satisfaction with a pharmacist-provided telephone medication therapy management program [23].

Anaesthetists and pharmacists demonstrated stronger agreement with statements relating to the impact and usefulness of pharmacist-generated medication lists when compared with nursing and midwifery staff. For the statement, “I find it useful having the medication list prior to assessing the patient”, 8/11 (72.7%) nursing and midwifery staff strongly agreed, compared with 11/12 (91.7%) of anaesthetists strongly agreeing and all pharmacists, 9/9 (100%), who strongly agreed with this statement. This may reflect the more direct integration of medication lists into anaesthetic assessment, perioperative medication charting, and pharmacist BPMH preparation workflows.

A small number of anaesthetists selected “neutral” in response to statements relating to the usefulness of written pharmacist recommendations regarding medication interactions with anaesthetic agents (2/12, 16.7%) and whether access to a PAC pharmacist supported the management of medication-related queries (1/12, 8.3%). Free-text responses suggested that anaesthetists placed greater value on the PAC pharmacist role in medication reconciliation and advice regarding medications requiring withholding prior to surgery, rather than detailed written recommendations regarding anaesthetic drug interactions.

Several limitations should be considered when interpreting the findings of this study. Participation in the staff survey was voluntary, introducing the potential for self-selection bias, which may limit generalizability of the results. Additionally, pharmacists were relatively new to the PAC clinic environment and the DMR system following formalization of the PAC pharmacist role. During the initial implementation phase, PAC pharmacist-completed medication lists were paper-based and scanned into the DMR, which may have reduced visibility and accessibility for other healthcare professionals, including ward pharmacists. This limitation was addressed through staff education and increased awareness regarding the location and utilisation of PAC pharmacist documentation within the DMR. This study also did not measure the proportion of patients with a completed BPMH, medication reconciliation, and home medications charted correctly within 24 h of admission, which limited the impact of the PAC pharmacist in medication reconciliation. This study also did not include a control group. The impact of the PAC pharmacist measured in the study centred on the perception of the impact, and was measured by surveying the clinical pharmacists on the usefulness of a medication list generated by a PAC in reducing the time required to complete a BPMH on admission, the improved workflow efficiency, and a reduction of charting errors on admission. Finally, this study was conducted at a single tertiary women’s hospital, and therefore findings may not be generalizable to other healthcare settings or patient populations outside specialized women’s health services.

## 5. Conclusions

Our study suggested that telephone consultation was effective for obtaining BPMHs and providing medication advice for most obstetric patients in a PAC setting. The new pharmacy-led service was recognized as valuable and beneficial by anaesthetists, nursing and midwifery staff, allied health staff, and pharmacists, with high levels of agreement regarding the usefulness of pharmacist involvement. Staff feedback suggested that the role improved workflow efficiency, enhanced preparedness for patient assessment, supported medication charting processes, and assisted in the management of medication-related queries within the PAC environment. Importantly, no negative responses were received regarding satisfaction with the service, further highlighting the strong multidisciplinary acceptance of the role.

## Figures and Tables

**Table 1:** Some examples of responses received.

Role	Responses
Nurse/midwife/allied health	Pharmacy is vital to the care of the patient prior to their procedure as well as recovery care. Their knowledge helps the anaesthetic team and nursing staff support the patient through there hospital journey.
Nurse/midwife/allied health	Having a consistent presence of a known pharmacist at all clinics is incredibly helpful, i.e., I really noticed it when [the regular pharmacist] was on leave and others were covering or especially when we had no cover!! It definitely helps having these assessments completed before we see the patients and the advice provided to the patients is incredibly valuable.
Nurse/midwife/allied health	PAC Pharmacy supports us very well in the PAC caesarean section clinic and it should be a permanent role.
Anaesthetist	It would be ideal if the medication advice could be a joint statement (i.e., discuss complex cases with anaesthetist and issue advice) currently it seems to be a 2-step process (1. See pharmacist 2. Anaesthetist gives final advice) I understand this might be logistically challenging
Anaesthetist	Works great very useful saves me a lot of time.
Anaesthetist	Rather than “interactions with anaesthetic agents” the utility of PAC pharmacist from our point of view is 1. reconciliation AND 2. consistency in messaging of medications needing to be withheld preop, and timing of that, as per the agreed PAC/anaesthetic guidelines.
Anaesthetist	Occasionally the medication advice (e.g., what to withhold) is overly conservative but that is subjective and I’d prefer more rather than less.
Anaesthetist	Having a consistent presence of a known pharmacist at all clinics is incredibly helpful, i.e., I really noticed it when our regular PAC pharmacist was on leave and others were covering or especially when we had no cover! It definitely helps having these assessments completed before we see the patients and the advice provided to the patients is incredibly valuable.
Pharmacist	Patients are having their regular medications charted on their arrival to the ward either pre or postop, when previously patients would get their regular medications charted only once I’ve seen them to do their MMP usually, the day after their surgery
Pharmacist	Extremely valuable service!
Pharmacist	Continue as is. Time saving if patient has interacted with PAC service when taking the BPMH on the wards
Pharmacist	Confirmation PAC pharmacy service is supported in the long term

## Data Availability

The original contributions presented in this study are included in the article. Further inquiries can be directed to the corresponding author.

## Abbreviations

The following abbreviations are used in this manuscript:

PAC	Pre-admission clinic
BPMH	Best possible medication histories
DMR	Digital medical record
